# Ultrasound-Based Techniques for Visualization of Dermal Microvasculature: A Scoping Review

**DOI:** 10.3390/diagnostics16101435

**Published:** 2026-05-08

**Authors:** Rikke Baarts, Alexander Cuculiza Henriksen, Nathalie Sarup Panduro, Emma Kanchana Ertner Bengtsson, Ali Salari, Caroline Clausen, Lisbet Rosenkrantz Hölmich, Lars Lönn, Charlotte Mehlin Sørensen, Jørgen Arendt Jensen, Michael Bachmann Nielsen

**Affiliations:** 1 Department of Clinical Medicine, University of Copenhagen, 2200 Copenhagen, Denmark; ahen0070@regionh.dk (A.C.H.); nathalie.sarup.panduro.01@regionh.dk (N.S.P.); emma.olivia.kanchana.ertner.bengtsson.01@regionh.dk (E.K.E.B.); caroline.clausen@regionh.dk (C.C.); lars.birger.loenn@regionh.dk (L.L.); 2 Department of Diagnostic Radiology, Rigshospitalet, 2100 Copenhagen, Denmark; cmehlin@sund.ku.dk; 3 Center for Fast Ultrasound Imaging (CFU), DTU Health Tech, 2800 Kgs Lyngby, Denmark; alisa@dtu.dk (A.S.); jaje@dtu.dk (J.A.J.); 4 Department of Plastic Surgery, Copenhagen University Hospital, Herlev and Gentofte, 2730 Herlev, Denmark; lisbet.rosenkrantz.hoelmich@regionh.dk; 5 Department of Biomedical Sciences, Division of Integrative Circulation, University of Copenhagen, 2200 Copenhagen, Denmark

**Keywords:** dermal microvasculature, ultrasound, photoacoustic imaging

## Abstract

**Objectives:** To systematically map the existing literature on ultrasound-based techniques for non-invasive visualization of the dermal microvasculature and identify methodological strengths, limitations, and evidence gaps. **Methods:** This scoping review was conducted according to PRISMA-ScR guidelines and registered on the Open Science Framework (DOI: 10.17605/OSF.IO/7VDUK). MEDLINE, PubMed, Embase, Scopus, and Web of Science were searched (January 2000–October 2025). Studies involving human participants and ultrasound-based techniques explicitly aimed at visualizing dermal microvasculature were included. Data on study design, population characteristics, imaging parameters, and reported outcomes were extracted and synthesized narratively. **Results:** Thirty-six studies published between 2007 and 2025 were included. Most were small feasibility or experimental studies (*n* = 24), with a median sample size of three participants and substantial heterogeneity in imaging protocols. Photoacoustic-based techniques were most frequently reported (*n* = 21) and were the most consistently described as providing high microvascular detail and functional assessment capability. High-frequency ultrasound (*n* = 10) and advanced Doppler methods (*n* = 7) also enabled visualization of dermal vessels, but showed variability in sensitivity, reporting, and standardization. Validation against histopathology was reported in only one study. **Conclusions:** Ultrasound-based techniques can visualize dermal microvasculature in vivo; however, evidence remains fragmented, methodologically heterogeneous, and largely derived from small exploratory studies. Standardized imaging protocols, pathology-based clinical cohorts and robust validation studies are required to establish comparative performance and enable clinical translation in radiology.

## 1. Introduction

Advances in dermatological imaging over the past decade have contributed to improved diagnostic accuracy and longitudinal assessment of cutaneous disease [1,2,3,4,5]. The ability to visualize the dermal microvasculature has increased with recent imaging developments. Microvascular changes are a key feature of multiple dermatological conditions, including melanoma, psoriasis, systemic sclerosis, chronic wounds, and neovascular remodeling in skin tumors [2,5,6,7,8]. Non-invasive imaging modalities offer advantages related to safety, patient comfort, and the ability to support early diagnosis and intervention [4,7,9].

Ultrasound, as a non-invasive, cost-effective, and readily accessible imaging modality, has emerged as a practical tool for evaluating cutaneous structures. Techniques such as high-frequency ultrasound (HFUS), Doppler ultrasound, and photoacoustic methods have improved the spatial resolution and sensitivity of dermal microvascular visualization; however, their adoption in routine hospital-based clinical practice remains limited [2,4,10,11,12,13]. The reliability, validity, and optimal protocols for ultrasound imaging of the dermal microvasculature have not yet been clearly established. A systematic mapping of existing evidence is therefore necessary to identify current capabilities, limitations, and evidence gaps to guide future research and clinical practice.

The primary aim was to systematically map the existing literature on ultrasound-based non-invasive imaging of the dermal microvasculature, with a focus on identifying methodological strengths, weaknesses, and gaps in current evidence. A secondary aim was to outline priorities for future research to improve diagnostic accuracy and clinical applicability.

## 2. Materials and Methods

We examined ultrasound-based visualization of the dermal microvasculature, focusing on reported technical parameters, their impact on image quality, and the ability to capture and quantify small dermal vessels. The influence of patient- and lesion-specific characteristics on visualization quality and accuracy was also assessed. Another central aim was to evaluate how effectively different ultrasound parameters capture and quantify the smallest dermal vessels. Finally, the review investigated whether patient demographics or lesion-specific characteristics influence the accuracy or quality of ultrasound-based visualization.

Studies involving human adult participants, both healthy and with dermatological conditions, were included. Only research that used ultrasound-based techniques explicitly aimed at visualizing dermal microvasculature (either visually defined as the smallest vessels within the dermis or measured as structures ≤ 100 µm) was considered. Eligible study types included original research, including clinical trials, cohort studies, and cross-sectional designs, as well as relevant review articles. Publications published between January 2000 and October 2025 in English were included.

Exclusion criteria included studies of non-dermal microvasculature, non-ultrasound modalities without ultrasound comparison, purely laboratory-based studies without clinical relevance, editorials or opinion pieces or letters without visualization of microvasculature, and studies lacking full-text availability.

### 2.1. Protocol Registration and Search Strategy

This scoping review was registered on the Open Science Framework (OSF) (No. 10.17605/OSF.IO/7VDUK) and conducted in accordance with the preferred reporting items for systematic reviews and meta-analysis extension for scoping reviews (PRISMA-ScR guidelines see Figure S1 in Supplementary Materials) [14].

A systematic search strategy was designed in collaboration with the Medical Library at Rigshospitalet, Copenhagen, employing a combination of relevant keywords and Medical Subject Headings (MeSH) terms. Databases searched included MEDLINE (Ovid), PubMed, Embase (Ovid), Scopus, and Web of Science, covering publications from January 2000 to January 2024. An update search was conducted on 9 October 2025 to include the most recent additions, and totals were updated accordingly. Reference lists from included articles were manually screened for additional studies. For a detailed view of the search strategy, see Appendix A.

### 2.2. Study Selection and Data Extraction

Identified studies were managed using Covidence software, with a total of 2777 records identified, 1563 duplicates removed, 1214 titles and abstracts screened, and 111 full texts assessed, resulting in 36 studies included in the review.

**Figure 1:**
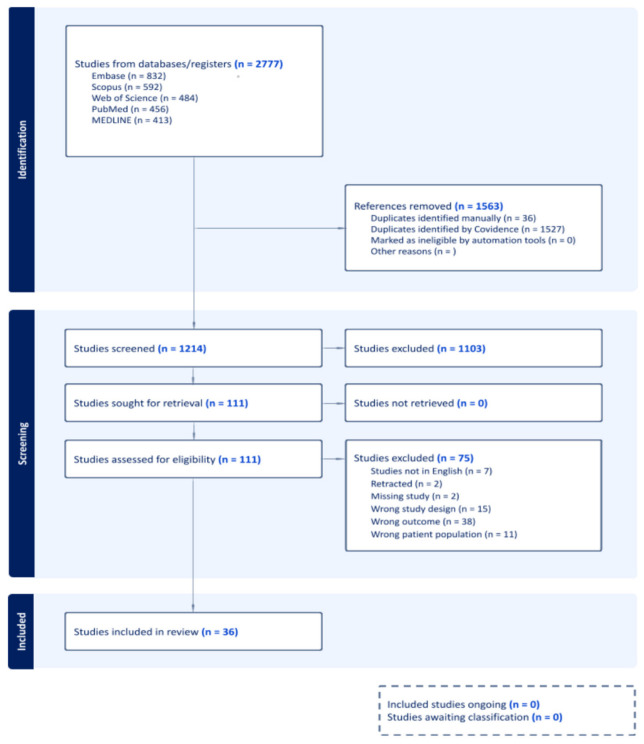
All studies were selected following the PRISMA guidelines, using the tools available in Covidence [14].

Titles and abstracts were screened independently by two reviewers, with discrepancies resolved by discussion and when necessary, a third senior reviewer. The study selection process was documented following PRISMA guidelines (Figure 1).

Data extraction utilized a standardized form, refined iteratively, capturing study design, demographics, ultrasound techniques and parameters, clinical context, and findings related to dermal microvascular visualization. Extraction was conducted in duplicate to minimize bias, and discrepancies in extracted items were resolved through discussion or a third senior reviewer. Data were synthesized narratively, highlighting methodological heterogeneity and technological variations. For the full data extraction, see Appendix B. For device-, probe-, transducer-, and software-level technical details reported across the included studies, see Appendix C.

Critical appraisal/risk-of-bias assessment was not performed, as this scoping review aimed to map the extent and characteristics of the evidence base in accordance with PRISMA-ScR/JBI guidance.

### 2.3. Deviations from Protocol

Several included studies utilized multimodal imaging, requiring careful extraction to isolate ultrasound-specific findings clearly. Inconsistent reporting of demographic and lesion characteristics limited the possibility of detailed subgroup analyses, influencing the comprehensiveness of synthesized results.

## 3. Results

### 3.1. Study Characteristics

Out of 2777 records, a total of 36 studies published between 2007 and 2025 were included. The vast majority were non-randomized experimental studies or feasibility studies (*n* = 24), which were primarily designed as proof-of-concept studies. Only nine studies used a formal clinical design (three case reports, two case series, two case-control studies, and two cohort studies). In addition, three papers were narrative, systematic reviews, or opinion articles. This distribution reflects that the field is still in an early and exploratory stage, with few studies embedded in robust clinical study frameworks. For the complete data extraction, see Appendix B.

#### 3.1.1. Ultrasound Techniques

**Figure 2:**
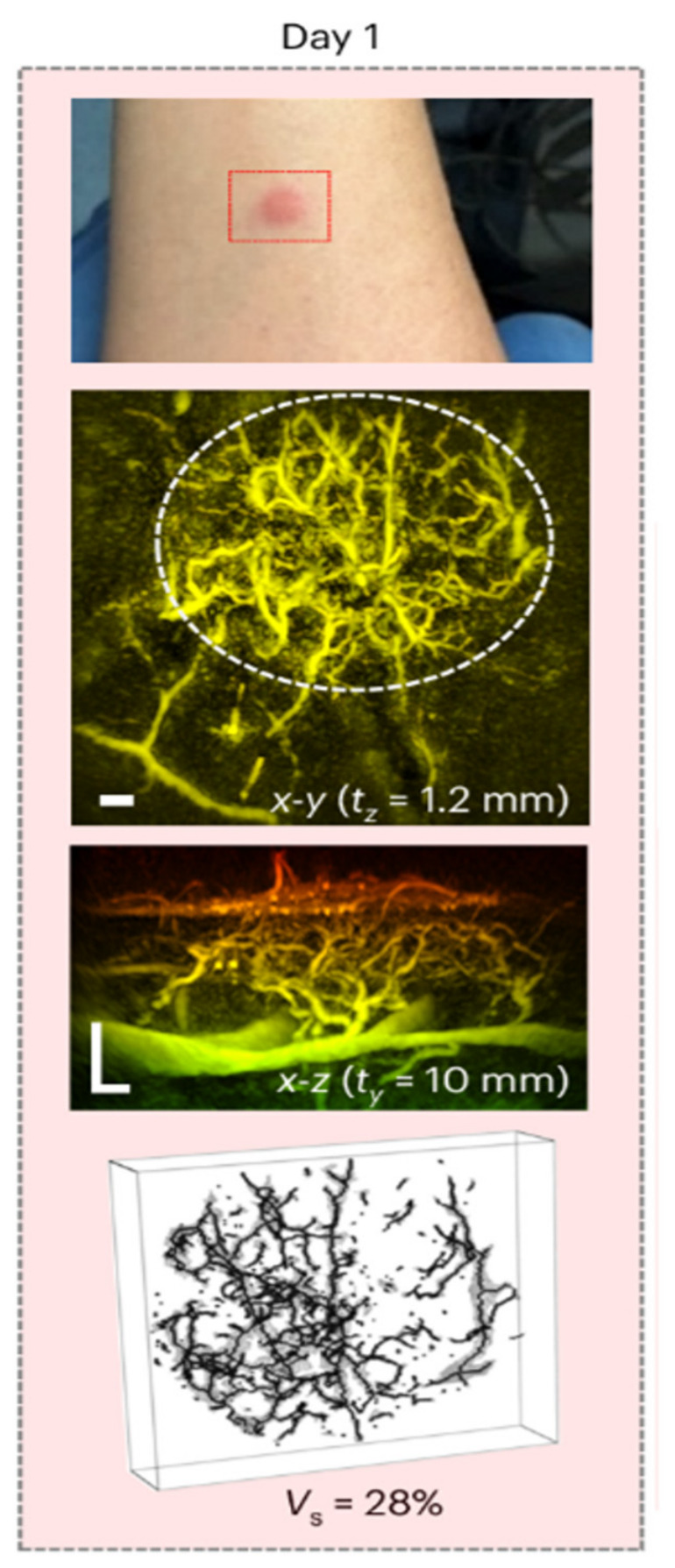
Photoacoustic imaging of inflammation around raised skin papule over 38 days. The lowest image shows a 3D skeleton representation of vascular architecture used to estimate vascular density, Vs, shown as a percentage. This image was published under open access, CC-BY 4.0 rights, and originates from [24].

A wide range of techniques were reported to visualize dermal microvasculature. Photoacoustic-based methods (including photoacoustic imaging, optical-resolution, photoacoustic-ultrasound dermascopy, photoacoustic tomography, and optoacoustic mesoscopy) were the most widely reported (*n* = 21). These studies primarily focused on technological innovation and demonstration of vascular detail, often at or below the level of the superficial dermal plexus, enabling visualization of the finest microvasculature and superficial vascular loops with penetration depths ranging from 1–5 mm (Figure 2) [8,15,16,17,18,19,20,21,22,23,24,25,26,27,28,29,30,31,32,33,34].

**Figure 3:**
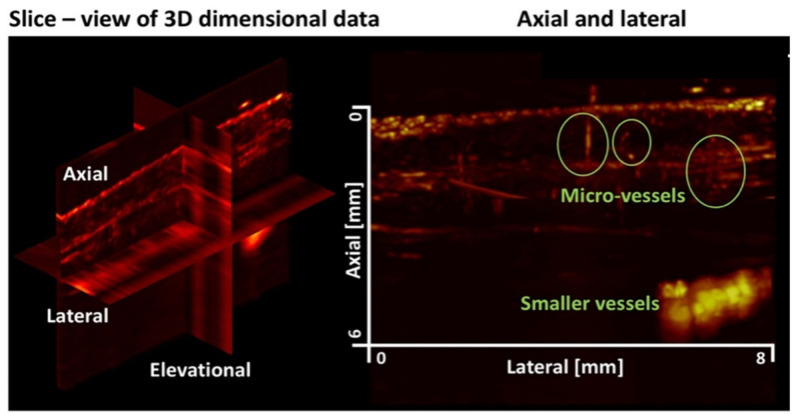
Using HFUS, the image shows volumetric visualization of dermal vasculatures on the dorsum of the hand with a new region-based SVD clutter filtering. Rendered in the slice-view and volumetric view to locate the vasculature along the volume in maximum intensity projection images. This image was published under open access, CC-BY 4.0 rights, and originates from Volumetric Visualization of the Dermal Vasculature with Signal and Image-based Feature Extraction on a High-frequency Ultrafast Ultrasound Dataset; [37] Seven studies reported the use of advanced Doppler-based techniques (SMI, MV-Flow, MicroV, and AP), describing enhanced sensitivity to low-flow velocities compared with conventional Doppler ultrasound, while noting limitations in visual representation relative to some other modalities (Figure 4) [9,10,11,35,36,40,41].

High-frequency ultrasound (HFUS) was used in 10 studies, typically in the range 20–70 MHz (Figure 3) [2,4,6,7,9,28,35,36,37,38,39]. In several of these, HFUS served as a structural reference modality without an independent visualization of the microvasculature. However, in five studies, it was used in combination with Doppler, providing visual information of the microvasculature [4,7,9,28,36]. One study by Catalano (2022) investigated three methods—HFUS, color Doppler, and superb microvascular imaging (SMI)—to visualize small dermal vessels in normal skin; however, the visual representation lacks the high resolution that some of the other modalities provide [9]. In 2025, one volumetric HF/ultrafast workflow demonstrated 3D dermal vascular mapping with region-based processing and image-feature extraction [37].

**Figure 4:**
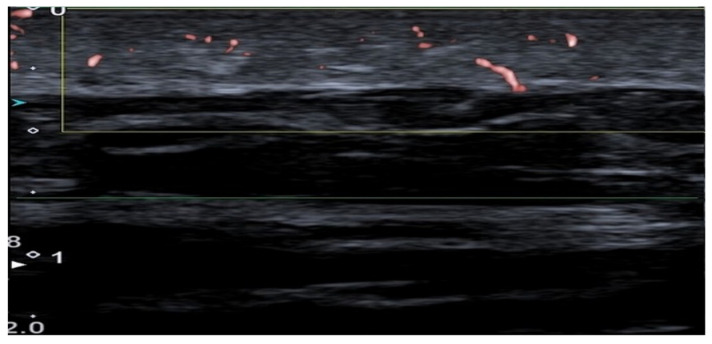
Superb Microvascular Imaging (SMI) of the dermal vascularization in the forearm (upper part of the image), with 1933 colored pixels, and with Power Doppler (lower part of the image), with 0 colored pixels. This image was published under open access, CC-BY 4.0 rights, and originates from [10].

**Figure 5:**
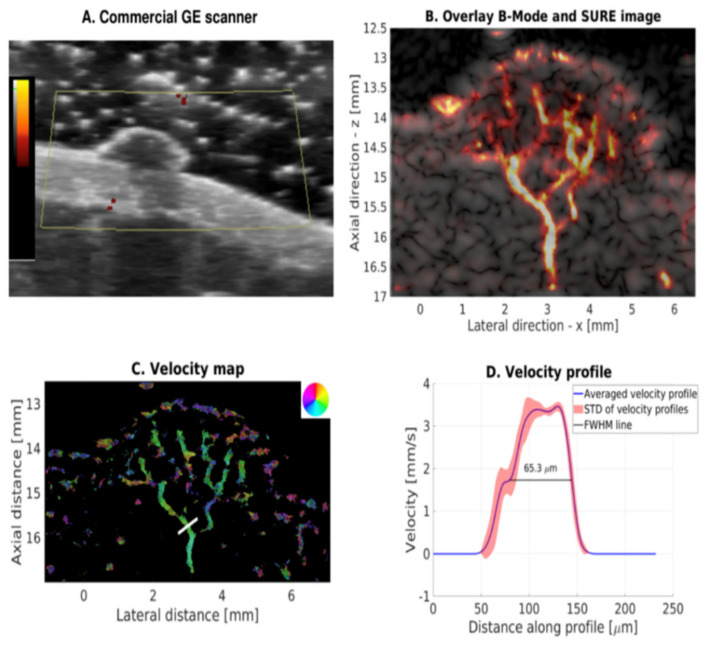
Super-resolution ultrasound imaging (SURE) of a benign cutaneous nevus in a healthy human subject. (**A**) Power Doppler image acquired using a commercial GE LOGIQ E9 ultrasound system. (**B**) Overlay of conventional B-mode ultrasound and SURE density imaging, illustrating the underlying microvascular architecture. (**C**) Corresponding microvascular velocity map. (**D**) Velocity profile extracted from the region of interest indicated in panel C. This figure is reproduced from an IEEE-published work with permission. Copyright © IEEE. Original source [12].

Other non-invasive ultrasonic methods include Super Resolution Ultrasound (SURE), a promising field rapidly in progress where tracking of the erythrocytes leads to highly detailed images with a spatial resolution of less than half a wavelength (Figure 5) [12].

Nine studies reported the combined use of two or more ultrasound-based modalities, most commonly HFUS with photoacoustic imaging, Doppler ultrasound, or SMI/MicroV [4,7,9,11,25,28,36,40,41], and furthermore, a 2025 multimodal ultrasound cohort in patients with keloid integrated B-mode, shear-wave elastography, and angiographic parameters for monitoring disease severity and treatment response [36].

#### 3.1.2. Clinical and Pathological Focus Areas

More than half (*n* = 19) of the studies were performed on the skin of healthy volunteers without a specific disease focus. The purpose of these studies was to establish baseline patterns, test technical concepts, or make direct comparisons between different methods [9,10,30]. Five studies investigated benign pigmented lesions and reported visualization of microvascular patterns in small nevi with high spatial resolution [12,16,17,21,22]. In addition, two studies focused on benign fibrotic lesions (keloid scars) [36,39], and five studies investigated inflammatory or vascular conditions, including plaque psoriasis, chronic venous disease, systemic sclerosis with Raynaud’s phenomenon, and burn depth assessment [11,21,24,38,41]. Four studies examined malignant skin neoplasms, primarily basal cell carcinoma, squamous cell carcinoma, and melanoma [2,21,35,40]. One further study included a subcutaneous tumor within a broader “general dermal imaging” context [15].

For a summarized overview of the articles, see Table 1.

#### 3.1.3. Key Findings

According to the included studies, ultrasound-based techniques were reported to enable visualization of the dermal microvasculature across all modality groups.

Photoacoustic methods were most consistently reported to provide high spatial detail and the possibility of functional measurements (e.g., oxygen saturation), but, despite being used in 21 studies, these techniques remain primarily experimental, with limited integration into routine clinical workflows [8,15,17,18,21,23,24,25,26,29,30].

HFUS and Doppler ultrasound were reported to provide structural and flow-related information, while demonstrating lower sensitivity to slow flow in the microvasculature compared with other ultrasound-based technique [3,6,38].

Several studies reported improved sensitivity to low-velocity dermal flow using advanced Doppler techniques such as SMI and MicroV compared with conventional Doppler ultrasound [9,10,11,41].

One study using SURE reported detailed microvascular imaging of a benign mole without the use of contrast agents [12]. While technically promising, evidence for dermal application of SURE is currently limited to this single study.

**Figure 6:**
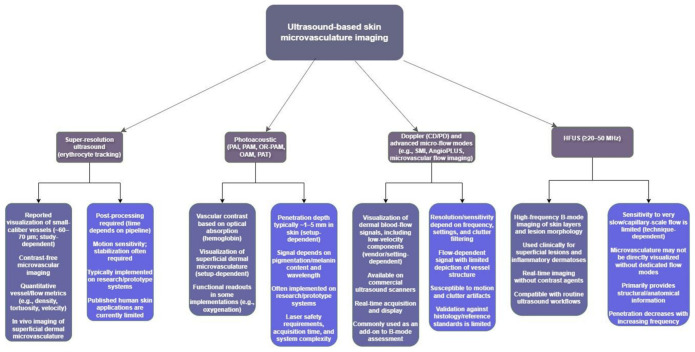
An overview of the reported strengths and limitations of the different modalities included in the study. Abbreviations: PAI, Photoacoustic Imaging; PAM, photoacoustic microscopy; PAT, photoacoustic tomography; OAM, optoacoustic mesoscopy; OR-PAM, Optical-Resolution Photoacoustic Microscopy; HFUS, high-frequency ultrasound; SMI, superb microvascular imaging; MV-Flow, microvascular flow imaging; MicroV, microvascular imaging; SURE, super-resolution ultrasound.

Please refer to Figure 6 and Table 2 for an overview of the different modalities, which provides a clear view of their reported accumulated strengths and weaknesses.

#### 3.1.4. Reporting Limitations

Overall, reporting quality and study size were limited. The median sample size was three participants, and, although sample size was clearly reported in most studies (*n* = 27), the majority enrolled fewer than 20 participants (*n* = 21). Demographic data were inconsistently reported and often not described. Nine studies did not clearly report their sample size. Demographic descriptors such as age (*n* = 13) and sex (*n* = 16) were frequently incomplete, and only seven studies had predefined inclusion and exclusion criteria prior to the study period.

Ultrasound protocols were heterogeneous, even within the same modality, with substantial variation in transducer frequency, imaging depth, frame rate, and reconstruction or postprocessing methods. Validation against reference standards was rarely described, with only a single study validating its findings against histopathology [17]. Technical barriers such as motion artifacts and limited depth penetration were mentioned as limitations in some studies [9,22,24,38]. A detailed overview of systems/platforms, probes/transducers, and reported technical settings is provided in Appendix C.

## 4. Discussion

Ultrasound-based visualization of the dermal microvasculature has advanced substantially over the past two decades, with recent improvements in spatial and temporal resolution enabling increasingly detailed assessment. Despite these technological gains, the current evidence base is fragmented and at an early stage. Most included studies are small feasibility investigations employing heterogeneous methods and reporting standards, with a median sample size of three participants, and frequent reliance on healthy volunteers rather than pathology-based cohorts. As a result, the clinical utility and comparative performance of these techniques remain uncertain, highlighting the need for standardized evaluation and larger, methodologically robust studies.

Photoacoustic techniques (Figure 2) were the most frequently represented modality group and showed the ability to provide detailed insight into the dermal microvasculature, and some studies have hypothesized the possibility to differentiate benign melanocytic tumors from malignant melanomas by the different vascular pattern parameters, including blood flow, tortuosity, and vessel density [8,15,17,18,21,23,24,25,26,29,30]. Acquisition time and penetration depth, as seen in the early generations of photoacoustic scans, were some of the troubles hindering the use in clinical practice; however, recently there have been successful scans with just a few seconds of acquisition time [24,34]. Schwartz et al. (2015) demonstrated that they can visualize vascular structures as deep as 2 mm with a frequency of 25 MHz [22]; however, with higher frequencies, and therefore details, the depth penetration significantly declines [19]. Clinical availability is still limited, though several studies are making progress in creating and using a hand-held photoacoustic probe. Thus, the research is still largely on experimental basis with larger studies warranted [16,24,27,29,30]. Another limitation discovered by Liu et al. (2016) in a study using photoacoustic tomography was skin type [31,32]. They categorize skin types by the Fitzpatrick scale of I–VI, where the groups are defined by the amount of pigment in the skin and the readiness of it to burn when exposed to direct sunlight [32,42]. The laser is absorbed by the superficial pigment in the skin, thus hindering penetration of the laser, and therefore the depth of the scan will increase with a decreasing amount of pigment in the skin [31,32]. This also applies to moles containing pigment. Schwarz et al. (2015) found that the pigment in a benign mole absorbed the laser and decreased the signal in the area of the mole, however, not hindering visualization of the microvasculature in the skin underneath [22]. More broadly, skin type was not reported consistently across the included studies, representing an important reporting gap, particularly for optical ultrasound techniques in which pigmentation may influence signal penetration and image interpretation.

Photoacoustic tomography is a field continuously researched, and new insights into the methods keep arising. Rodrigues et al. (2022) found that multi-spectral optoacoustic imaging (MSOT) could differentiate physiological parameters in the skin, such as HbO2, Hb, HbT, and mSO2 (MSOT-derived oxygen saturation) [33]. They argue that MSOT can be used as a predictive measure in clinical practice; however, they clearly state that their findings are regarded as exploratory and should be followed by larger clinical studies [33]. Another study by Zheng et al. (2021) argues that they can improve biometric security of fingerprinting by visualization of the underlying microvasculature of the fingertips [26].

HFUS (Figure 3) is widespread and readily available, but generally lacks resolution to visualize the capillary level [2,38]. However, a study by Chen et al. (2017), and more recently by Zhou et al. (2025), showed promising results using a 40 MHz linear probe to visualize the microvasculature in a keloid scar [36,39]. A study by Bhatti et al. (2023) was successful in showing the microvasculature of the dermal layers when HFUS is filtered with region-based SVD and top hat filtering [6]. An automated workflow for processing, using an ultra-high frequency probe (>40 MHz) and super-resolution imaging techniques, was their suggestion for future improvements to visualize microvasculature in the skin [6].

Advanced Doppler methods (SMI, MicroV, MV-Flow, AP), as seen in Figure 4, show improved ability to visualize microvasculature in the skin and could possibly be the first to find their way into dermatological practice due to their widespread availability and high resolution [9,10,11,35,41]. Jasionyte et al. (2023) used a combination of high-resolution ultrasound and SMI to visualize dermal microvascular flow, using a new filtering system that minimizes motion artifacts [35]. They argue that the method can be used in the discrimination of ultrasonic patterns in patients with systemic sclerosis, as the vascular changes appear to be prominent and obvious, but as with many of the studies, it is limited by a small sample size [11,35]. The evidence for identifying malignant melanoma using SMI is sparse and based on very small series. Kho et al. (2024) indicated that vascular patterns can support differentiation of tumors, but the material is still too sparse for clinical conclusions, and the studies are too small in size [40]. A single study using SURE provided highly detailed microvascular images of a benign mole, including flow measurements of 3.2 mm/s and estimated vessel measurements of 65 µm in diameter [12]. Al-though technically promising, dermal application of SURE remains at a very early stage and its clinical relevance cannot yet be inferred from a single included study (Figure 5) [12,43].

Other modalities, such as laser Doppler imaging and laser speckle contrast imaging, have given important insights into the dermal microvasculature by providing high-resolution depictions of vascular flow and morphology at superficial depths [44,45]. Photoacoustic imaging combines pulsed laser excitation with ultrasound detection, thereby serving as a technical bridge between optical contrast and ultrasonic penetration [19,32].

While the current studies remain largely exploratory, several potential clinical uses can be identified based on the included studies. In the context of pigmented lesions, microvascular imaging may serve as a diagnostic tool where differences in vascular architecture and flow patterns could support differentiation and delineation between benign nevi and malignant melanomas [2,12,17,21,35,40]. In inflammatory diseases such as psoriasis and systemic sclerosis, as well as fibrotic skin conditions, these techniques could offer a non-invasive approach to monitoring disease activity and treatment response through changes in microvascular density and perfusion [11,21,24,35,36,39]. Furthermore, preliminary findings suggest a potential role in burn depth assessment, where evaluation of dermal perfusion may support clinical decisions [38]. While interesting and promising, these potential applications remain insufficiently validated.

Across studies, common limitations included non-standardized imaging and analysis protocols, small and homogeneous populations, frequent use of healthy volunteers, and technical challenges such as motion artifacts and restricted imaging depth, all of which may limit the generalizability and reliability of findings. Notably, validation against a clinical reference standard was largely absent, with only one included study reporting histopathological confirmation. This represents a critical evidence gap, as biopsy-correlated studies are essential if these technologies are to move from technical feasibility toward clinically meaningful diagnostic applications.

An additional key challenge, highlighted in the technical overview (Figure 6 and Appendix C), is the substantial heterogeneity not only across imaging modalities, but also within them. Even within commonly used approaches such as high-frequency ultrasound and advanced Doppler techniques, e.g., SMI, there was a notable variation in the used systems, probes, frequencies, and processing methods, which further limits comparability between studies.

Clinical translation will require not only standardized image acquisition, reporting, and quantification, but also greater consistency in the technical implementation across modalities. Future research should prioritize adequately powered pathology-based cohorts and multicenter study designs, including direct comparison of modality, and biopsy-correlated validation studies to establish reliability, generalizability, and clinical relevance.

## 5. Conclusions

Ultrasound-based techniques can visualize the dermal microvasculature in vivo, but evidence is limited, fragmented, and largely restricted to small exploratory studies with heterogeneous protocols. Among the included studies, photoacoustic-based methods most consistently reported high microvascular detail, whereas advanced Doppler techniques may represent one of the more clinically accessible approaches in the near term. High-frequency ultrasound remains structurally useful but is generally insufficient for capillary-level assessment without specialized processing. Standardized imaging protocols, robust validation, and adequately powered pathology-based studies are needed to determine comparative performance and support clinical translation. With standardized methods and clinically grounded validation, ultrasound-based microvascular imaging may evolve from a technical innovation into a more clinically relevant tool in dermatology.

## Figures and Tables

**Table 1:** Summarized overview of the preferred methods, focus areas, and reporting variabilities in the included studies. Note that some studies utilized more than one method. Abbreviations: CDFI, Color Doppler Flow Imaging; HFUS, high-frequency ultrasound; MicroV, microvascular imaging; MV-Flow, microvascular flow imaging; *n*, number; OAM, optoacoustic mesoscopy; PAI, photoacoustic imaging; PAM, photoacoustic microscopy; PAT, photoacoustic tomography; PDI, Power Doppler Imaging; SMI, Superb Microvascular Imaging; VHFUS, very high-frequency ultrasound.

Modality	Number of Studies
Photoacoustic-based (PAI/PAM/PAT/OAM)	21
High-frequency ultrasound (HFUS/VHFUS)	10
Conventional Doppler (CDFI/PDI)	7
Advanced Doppler (SMI/MicroV/MV-Flow)	7
Multimodal combinations (≥2 modalities)	9
Super-Resolution Ultrasound using tracking of the erythrocytes	1
Clinical and pathological focus areas	
Healthy volunteers/normal skin	19
Inflammatory/vascular disease	5
Benign fibrotic lesions (keloid)	2
Benign pigmented lesions (nevi/moles)	5
Malignant skin tumours	4
Other pathology	1
Reported variabilities	
Median sample size (participants)	3
Studies < 20 participants	21
Studies missing numeric sample size	9
Studies reporting age	13
Studies reporting sex	16
Studies reporting both inclusion and exclusion criteria	7
Studies informing about histopathology	1

**Table 2:** Summary of reported strengths, limitations, and clinical status of the included ultrasound-based modalities for dermal microvascular imaging. Some studies used more than one modality. Abbreviations: AP, AngioPLUS; CDFI, color Doppler flow imaging; HFUS, high-frequency ultrasound; PDI, power Doppler imaging; SMI, superb microvascular imaging; SURE, super-resolution ultrasound using erythrocytes; VHFUS, very high-frequency ultrasound.

Modality	Studies (*n*)	Main Strengths	Main Limitations	Clinical Status
Photoacoustic-based imaging	21	High microvascular detail; functional imaging possible	Mostly experimental; limited standardization; depth–resolution trade-off; affected by pigmentation/skin type	Mainly experimental
High-frequency ultrasound (HFUS/VHFUS)	10	Widely available; useful structural imaging	Limited capillary-level detail without added processing	Clinically available, but limited for fine microvascular imaging
Conventional Doppler (CDFI/PDI)	7	Flow-related information; widely available	Low sensitivity to slow dermal flow; limited fine-vessel depiction	Clinically established, but limited for dermal microvasculature
Advanced Doppler (SMI/MicroV/MV-Flow/AP)	7	Better sensitivity to low-flow dermal vessels; relatively accessible clinically	Small heterogeneous evidence base; device-dependent; lower detail than highest-resolution techniques	Promising clinical adjunct
Super-resolution ultrasound using erythrocytes (SURE)	1	Very high spatial detail; contrast-free microvascular imaging	Only one included dermal study; no basis yet for clinical readiness	Very early-stage

## Data Availability

The original contributions presented in this study are included in the article/Supplementary Material. Further inquiries can be directed to the corresponding authors.

## Supplementary Materials

The following supporting information can be downloaded at: https://www.mdpi.com/article/10.3390/diagnostics16101435/s1, Figure S1: Preferred Reporting Items for Systematic reviews and Meta-Analyses extension for Scoping Reviews (PRISMA-ScR) Checklist.

## Abbreviations

AP	Angiography Mode (advanced Doppler angiographic mode)
AR-PAM	Acoustic-Resolution Photoacoustic Microscopy
BCC	Basal Cell Carcinoma
B-mode	Brightness Mode ultrasound
CFU	Center for Fast Ultrasound Imaging
CDI	Color Doppler Imaging
CDFI	Color Doppler Flow Imaging
Doppler US	Doppler Ultrasound
Hb	Deoxygenated Hemoglobin
HbO_2_	Oxygenated Hemoglobin
HbT	Total Hemoglobin
HFUS	High-Frequency Ultrasound
MeSH	Medical Subject Headings
MIP	Maximum Intensity Projection
MSOT	Multispectral Optoacoustic Tomography
mSO_2_	Optoacoustic-derived oxygen saturation
MV-Flow	Microvascular Flow imaging (advanced Doppler technique)
MicroV	Microvascular Imaging
OR-PAM	Optical-Resolution Photoacoustic Microscopy
OAM	Optoacoustic Mesoscopy
OCT	Optical Coherence Tomography
OSF	Open Science Framework
PA	Photoacoustic
PAI	Photoacoustic Imaging
PAM	Photoacoustic Microscopy
PAT	Photoacoustic Tomography
PAUS	Photoacoustic and Ultrasound imaging
PDI	Power Doppler Imaging
PRISMA-ScR	Preferred Reporting Items for Systematic Reviews and Meta-Analyses extension for Scoping Reviews
ROI	Region of Interest
SCC	Squamous Cell Carcinoma
SVD	Singular Value Decomposition
SMI	Superb Microvascular Imaging
SURE	Super-Resolution Ultrasound using Erythrocytes
SWE	Shear Wave Elastography
US	Ultrasound
VHFUS	Very High-Frequency Ultrasound
Vs	Vascular density parameter

## Appendix A. Search Strategy

The inclusion criteria for this scoping review are based on the PCC framework:

Participants: Individuals who have been scanned on the skin with ultrasound, with no restrictions on disease, age, or gender.

Concept: The review will focus on ultrasound’s ability to visualize and quantify the dermal microvasculature. Studies involving other imaging techniques will be excluded unless a direct comparison with ultrasound is made.

Context: The review will include articles in clinical or research setting where imaging is performed, with no language, geographic, or healthcare system restrictions.

This broad inclusion ensures that the review captures the full scope of available evidence on the use of ultrasound in melanoma diagnostics, enhancing its generalizability. The latest search was conducted on the 9th of October 2025.

Search via Pubmed

(“Skin”[MeSH Terms] OR “Dermis”[MeSH Terms] OR “Epidermis”[MeSH Terms] OR skin*[Title/Abstract] OR derm*[Title/Abstract] OR cutan*[Title/Abstract] OR cutis*[Title/Abstract] OR mole[Title/Abstract] OR moles[Title/Abstract] OR nevi[Title/Abstract] OR naevi[Title/Abstract] OR nevus[Title/Abstract] OR melanoma*[Title/Abstract])

AND

((“Microvessels”[MeSH Terms] OR “Microcirculation”[MeSH Terms] OR “Microvascular Density”[Title/Abstract] OR microvascula*[Title/Abstract] OR microvessel*[Title/Abstract])

AND

(“Ultrasound Localization Microscopy”[Title/Abstract] OR ULM[Title/Abstract] OR “Super-Resolution Ultrasound Imaging”[Title/Abstract] OR SRI[Title/Abstract] OR SURE[Title/Abstract] OR “Localization-Based Ultrasound Imaging”[Title/Abstract] OR “Contrast-Enhanced Ultrasound”[Title/Abstract] OR CEUS[Title/Abstract] OR “Superb Microvascular Imaging”[Title/Abstract] OR SMI[Title/Abstract] OR “Ultrafast Imaging”[Title/Abstract] OR ultrasound[Title/Abstract] OR “Plane-Wave Imaging”[Title/Abstract] OR “Fast Ultrasound”[Title/Abstract] OR “Ultrasonography”[MeSH Terms]))

Search via Medline (Ovid)

**Set**	**Search Statement**
1.	exp microvessels/
2.	microcirculation/or microvascular density/
3.	skin/or dermis/or epidermis/
4.	(skin* or derm* or cutan* or cutis* or mole or moles or nevi or naevi or nevus or melanoma*).ab,kf,ti.
5.	(“ultrasound localization microscopy” or ULM or “super-resolution ultrasound imaging*” or “SRI” or “SURE” or “super-resolution ultrasound localization*” or “localization-based ultrasound imaging” or “microscale ultrasound imaging” or “ultrasound microvascular imaging*” or “super-resolution vascular ultrasound” or “high-resolution ultrasound microscopy” or “microbubble-enhanced ultrasound imaging*” or “ultrasound molecular imaging*” or “microbubble ultrasound localization microscopy*” or “vascular ultrasound localization imaging” or “super-resolution microvessel imaging” or “contrast-enhanced ultrasound*” or “CEUS” or “super-resolved ultrasound*” or “high-resolution microvascular imaging*” or “advanced microvascular imaging” or “superb microvascular imaging” or “SMI”).ab,kf,ti.
6.	(microvascula* or microvessel*).ab,kf,ti.
7.	(skin* or dermis or cutan* or cutis*).ab,kf,ti.
8.	ultrasonography/or elasticity imaging techniques/or microscopy, acoustic/or ultrasonography, Doppler/
9.	(“Ultrafast imaging” or ultrasound or “Plane-wave imaging” or “Fast ultrasound imaging”).ab,kf,ti.
10.	1 or 2 or 6
11.	3 or 4 or 7
12.	5 or 8 or 9
13.	10 and 11 and 12

P:

skin/or dermis/or epidermis/

(skin* or derm* or cutan* or cutis* or mole or moles or nevi or naevi or nevus or melanoma*).ab,kf,ti.

(skin* or dermis or cutan* or cutis*).ab,kf,ti.

C:

exp microvessels/

microcirculation/or microvascular density/

(microvascula* or microvessel*).ab,kf,ti.

C:

(“ultrasound localization microscopy” or ULM or “super-resolution ultrasound imaging*” or “SRI” or “SURE” or “super-resolution ultrasound localization*” or “localization-based ultrasound imaging” or “microscale ultrasound imaging” or “ultrasound microvascular imaging*” or “super-resolution vascular ultrasound” or “high-resolution ultrasound microscopy” or “microbubble-enhanced ultrasound imaging*” or “ultrasound molecular imaging*” or “microbubble ultrasound localization microscopy*” or “vascular ultrasound localization imaging” or “super-resolution microvessel imaging” or “contrast-enhanced ultrasound*” or “CEUS” or “super-resolved ultrasound*” or “high-resolution microvascular imaging*” or “advanced microvascular imaging” or “superb microvascular imaging” or “SMI”).ab,kf,ti.

ultrasonography/or elasticity imaging techniques/or microscopy, acoustic/or ultrasonography, doppler/

(“Ultrafast imaging” or ultrasound or “Plane-wave imaging” or “Fast ultrasound imaging”).ab,kf,ti.

Search via Embase (Ovid)

**1.**	**(ultrasonography or elasticity imaging or microscopy, acoustic or ultrasonography, doppler).ab,fx,hw,kf,ot,ti.**
2.	(ultrasound or “ultrafast imaging” or “plane-wave imaging” or “fast ultrasound imaging”).ab,fx,hw,kf,ot,ti.
3.	(“ultrasound localization microscopy” or ULM or “super-resolution ultrasound imaging*” or SRI or SURE or “localization-based ultrasound imaging” or “microscale ultrasound imaging” or “ultrasound microvascular imaging*” or “super-resolution vascular ultrasound” or “high-resolution ultrasound microscopy” or “microbubble-enhanced ultrasound imaging*” or “ultrasound molecular imaging*” or “microbubble ultrasound localization microscopy*” or “vascular ultrasound localization imaging” or “super-resolution microvessel imaging” or “contrast-enhanced ultrasound*” or CEUS or “super-resolved” or “advanced microvascular imaging” or “superb microvascular imaging” or SMI).ab,fx,hw,kf,ot,ti.
4.	(skin* or derm* or cutan* or cutis* or mole or moles or nevi or naevi or nevus or melanoma*).ab,fx,hw,kf,ot,ti.
5.	(microcirculation or “microvascular density”).ab,fx,hw,kf,ot,ti.
6.	(microvessels or microvascula* or microvessel*).ab,fx,hw,kf,ot,ti.
7.	exp microvascular density/
8.	skin blood flow/or skin carcinogenesis/or skin carcinoma/or skin blood vessel/or skin capillary/or skin/
9.	microcirculation/
10.	5 or 6 or 7 or 9
11.	4 or 8
12.	1 or 2 or 3
13.	10 and 11 and 12

P:

(skin* or derm* or cutan* or cutis* or mole or moles or nevi or naevi or nevus or melanoma*).ab,fx,hw,kf,ot,ti.

skin blood flow/or skin carcinogenesis/or skin carcinoma/or skin blood vessel/or skin capillary/or skin/

C:

microcirculation/exp microvascular density/(microcirculation or “microvascular density”).ab,fx,hw,kf,ot,ti. (microvessels or microvascula* or microvessel*).ab,fx,hw,kf,ot,ti.

C:

(ultrasonography or elasticity imaging or microscopy, acoustic or ultrasonography, doppler).ab,fx,hw,kf,ot,ti.

(ultrasound or “ultrafast imaging” or “plane-wave imaging” or “fast ultrasound imaging”).ab,fx,hw,kf,ot,ti.

(“ultrasound localization microscopy” or ULM or “super-resolution ultrasound imaging*” or SRI or SURE or “localization-based ultrasound imaging” or “microscale ultrasound imaging” or “ultrasound microvascular imaging*” or “super-resolution vascular ultrasound” or “high-resolution ultrasound microscopy” or “microbubble-enhanced ultrasound imaging*” or “ultrasound molecular imaging*” or “microbubble ultrasound localization microscopy*” or “vascular ultrasound localization imaging” or “super-resolution microvessel imaging” or “contrast-enhanced ultrasound*” or CEUS or “super-resolved” or “advanced microvascular imaging” or “superb microvascular imaging” or SMI).ab,fx,hw,kf,ot,ti.

Search via SCOPUS–584 hits d. 12.12.24

The second search was done on the 9th of October 2025, and range was set to all new articles in 2024 and 2025. This provided two addictional references.

TITLE-ABS-KEY(“Skin” OR “Dermis” OR “Epidermis” OR “skin*” OR “derm*” OR “cutan*” OR “cutis*” OR “mole” OR “moles” OR “nevi” OR “naevi” OR “nevus” OR “melanoma*”)

AND

TITLE-ABS-KEY(“Microvessels” OR “Microcirculation” OR “Microvascular Density” OR “microvascula*” OR “microvessel*”)

AND

TITLE-ABS-KEY(“Ultrasound Localization Microscopy” OR “ULM” OR “Super-Resolution Ultrasound Imaging” OR “SRI” OR “SURE” OR “Localization-Based Ultrasound Imaging” OR “Contrast-Enhanced Ultrasound” OR “CEUS” OR “Superb Microvascular Imaging” OR “SMI” OR “Ultrafast Imaging” OR “ultrasound” OR “Plane-Wave Imaging” OR “Fast Ultrasound”)

Search via Web of Science

TS = (“skin” OR “dermis” OR “epidermis” OR “skin*” OR “derm*” OR “cutan*” OR “cutis*” OR “mole” OR “moles” OR “nevi” OR “naevi” OR “nevus” OR “melanoma*”)

AND TS = (“microvessels” OR “microcirculation” OR “microvascular density” OR “microvascula*” OR “microvessel*”)

AND TS = (“ultrasound localization microscopy” OR “ULM” OR “super-resolution ultrasound imaging*” OR “SRI” OR “SURE” OR “super-resolution ultrasound localization*” OR “localization-based ultrasound imaging” OR “microscale ultrasound imaging” OR “ultrasound microvascular imaging*” OR “super-resolution vascular ultrasound” OR “high-resolution ultrasound microscopy” OR “microbubble-enhanced ultrasound imaging*” OR “ultrasound molecular imaging*” OR “microbubble ultrasound localization microscopy*” OR “vascular ultrasound localization imaging” OR “super-resolution microvessel imaging” OR “contrast-enhanced ultrasound*” OR “CEUS” OR “super-resolved ultrasound*” OR “high-resolution microvascular imaging*” OR “advanced microvascular imaging” OR “superb microvascular imaging” OR “SMI” OR “ultrasonography” OR “elasticity imaging techniques” OR “microscopy, acoustic” OR “ultrasonography, doppler” OR “Ultrafast imaging” OR “ultrasound” OR “Plane-wave imaging” OR “Fast ultrasound imaging”)

## Appendix B

**Study** **No.**	**Title**	**Author**	**Year of** **Publication**	**Country in Which the Study** **Conducted**	**Study Design**	**Population Description**	**Sample Size**	**Method of Intervention**	**Health Focus** **(e.g., Melanoma)**	**Conclusions**
1	Combined ultrasound and photoacoustic system for real-time high-contrast imaging using a linear array transducer	Jaeger et al. [8]	2017	Switzerland	Technology development and experimental proof-of-concept	Healthy volunteer	1	Photoacoustic-based	Normal skin	A combined US/PA linear-array system visualized microvascular structures in real time with high contrast. Demonstrates technical feasibility; clinical applications were proposed but not evaluated.
2	Photoacoustic and ultrasound [PAUS) dermoscope with high sensitivity and penetration depth by using a bimorph transducer	Wang et al. [15]	2020	China	Non-randomized experimental study	Healthy volunteer	1	Photoacoustic-based	General dermal imaging	A PA/US dermoscope prototype improved sensitivity and penetration for vascular imaging in skin. Microvascular visualization is technically promising, but clinical performance was not evaluated.
3	Handheld optical-resolution photoacoustic microscopy	Lin et al. [16]	2017	United States	Non-randomized experimental study	Healthy volunteer	1	Photoacoustic-based	General dermal microvasculature. Mole, cuticle, and normal skin	A handheld OR-PAM system visualized superficial microvasculature with high resolution in a human case. Feasibility is strong, but clinical usefulness and diagnostic performance remain untested.
4	In vivo photoacoustic microscopy of human cutaneous microvasculature and a nevus	Favazza et al. [17]	2011	United States	Non-randomized experimental study	Healthy volunteers	3	Photoacoustic-based	Pigmented lesion (nevus) and melanoma	Photoacoustic microscopy visualized detailed dermal microvasculature in normal skin and a nevus. Strong microvascular depiction, but diagnostic performance for lesions (including melanoma) was not assessed. Histopathologic confirmation.
5	Full-view in vivo skin and blood vessels profile segmentation in photoacoustic imaging based on deep learning	Ly et al. [18]	2022	Republic of Korea	Non-randomized experimental study	Healthy volunteer	1	Photoacoustic-based	Normal skin and subcutaneous vascular structures	Photoacoustic imaging visualized skin/subcutaneous vasculature and deep-learning improved vessel profile extraction. Useful for analysis automation, but clinical benefit and generalizability are not yet demonstrated.
6	Linear-array-based photoacoustic imaging of human microcirculation with a range of high frequency transducer probes	Zafar et al. [19]	2015	Ireland	Non-randomized experimental study	Healthy volunteer	Not clearly reported	Photoacoustic-based	Normal skin	Linear-array photoacoustic imaging produced 3D microcirculation images in human skin, with performance dependent on probe choice. Feasibility is shown, but clinical utility and reproducibility were not tested.
7	Photoacoustic imaging of the human forearm using 40 MHz linear-array transducer	Zafar et al. [20]	2014	Ireland	Case report	Healthy volunteer	1	Photoacoustic-based	Normal skin	A 40 MHz linear-array photoacoustic setup visualized superficial skin microvasculature in a healthy volunteer. Feasibility only; no clinical validation or comparative performance assessment.
8	Fast raster-scan optoacoustic mesoscopy enables assessment of human melanoma microvasculature in vivo	He et al. [21]	2022	Germany	Case control study	Healthy volunteers and patients	22	Photoacoustic-based	Normal skin, psoriasis, melanomas, and benign nevi	Optoacoustic mesoscopy visualized and quantified microvascular differences between melanoma and benign pigmented lesions. Potentially useful as an adjunct for differentiation, but requires validation in larger, clinically integrated studies.
9	Implications of ultrasound frequency in optoacoustic mesoscopy of the skin	Schwarz et al. [22]	2015	Germany	Non-randomized experimental study	Healthy volunteer	1	Photoacoustic-based	Normal skin (glabrous and hairy skin) and benign nevus	Frequency selection strongly affected depiction of dermal microvasculature and superficial layers in optoacoustic mesoscopy. Useful as methodological guidance for optimizing microvascular visibility, not as clinical evidence.
10	Design of a high-frequency array based photoacoustic microscopy system for micro-vascular imaging	Bitton et al. [23]	2007	United States	Non-randomized experimental study	Healthy volunteer	1	Photoacoustic-based	Normal skin	An early high-frequency array PA system imaged microvessels in human skin (reported <100 µm). Demonstrates feasibility only and has minimal clinical applicability without further validation.
11	A fast all-optical 3D photoacoustic scanner for clinical vascular imaging	Huynh et al. [24]	2024	UK	Non-randomized experimental study	Healthy volunteers and patients	Not clearly reported	Photoacoustic-based	Skin inflammation, diabetes, rheumatoid arthritis	The system enabled rapid 3D visualization of vessels (including deeper structures) with reduced motion artifacts. Microvasculature is clearly visible and clinically promising, but current evidence remains exploratory with limited validation.
12	Photoacoustic tomography of fingerprint and underlying vasculature for improved biometric identification	Zheng et al. [26]	2021	United States	Non-randomized experimental study	Healthy volunteer	1	Photoacoustic-based	Fingerprint	Photoacoustic imaging visualized subsurface vasculature beneath fingerprint patterns to support biometric identification. Vascular structures are visible, but this is not a dermatologic diagnostic application.
13	Miniaturized photoacoustic probe for in vivo imaging of subcutaneous microvessels within human skin	Zhang et al. [27]	2019	China	Non-randomized experimental study	Healthy volunteers	Not reported	Photoacoustic-based	General microvascular visualization in skin	A miniaturized PA probe visualized cutaneous/subcutaneous microvessels with micrometer-scale resolution and millimeter penetration (lateral resolution of ~8.9 µm and imaging depth of ~2.4 mm). Strong technical feasibility for microvessel depiction, but no clinical or pathological validation.
14	In vivo photoacoustic microscopy of human cuticle microvasculature with single-cell resolution	Hsu et al. [29]	2016	United States	Non-randomized experimental study	Healthy volunteers	9	Photoacoustic-based	Normal skin (microvasculature)	OR-PAM visualized capillary loops and even individual RBC-related signals, enabling functional hemodynamic readouts. Highly effective for microvascular depiction, but clinical utility in disease remains untested.
15	Visualization of vasculature using a hand-held photoacoustic probe: phantom and in vivo validation	Heres et al. [30]	2017	Netherlands	Non-randomized experimental study	Healthy volunteers	4	Photoacoustic-based	General vascular visualization	A handheld photoacoustic probe visualized superficial dermal vasculature in vivo in real time. Demonstrates feasibility for bedside vascular imaging, but clinical applicability and quantitative validation remain limited.
16	Noninvasive and high-resolving photoacoustic dermoscopy of human skin	Xu et al. [31]	2016	China	Non-randomized experimental study	Not reported	Not reported	Photoacoustic-based	Normal skin	Photoacoustic imaging differentiated microvascular patterns between port-wine stain lesions and normal skin (e.g., larger/deeper vessels in lesions). Potentially useful for lesion characterization/monitoring, but not validated against histology.
17	Combined multi-modal photoacoustic tomography, optical coherence tomography [OCT), and OCT angiography system with an articulated probe for in vivo human skin structure and vasculature imaging	Liu et al. [32]	2016	Austria	Non-randomized experimental study	Healthy volunteers	Not reported	Photoacoustic-based	Normal skin, nevus	Multimodal imaging visualized dermal microvasculature and skin structure, showing complementary vascular/morphologic information. Feasibility is demonstrated, but clinical performance and diagnostic value were not evaluated.
18	Optoacoustic imaging offers new insights into in vivo human skin vascular physiology	Rodrigues et al. [33]	2022	Portugal	Non-randomized experimental study	Healthy volunteers	10	Photoacoustic-based	Normal skin with reactive hyperemia after inflation of a cuff to occlude the brachial artery	Optoacoustic imaging captured depth-dependent microvascular perfusion dynamics during occlusion and reactive hyperemia. Useful for functional/physiological assessment, but tested only in healthy volunteers and without outcome validation.
19	Ultrasound array photoacoustic microscopy for dynamic in vivo 3D imaging	Song et al. [34]	2010	United States	Non-randomized experimental study	Healthy volunteers	1	Photoacoustic-based	Normal skin	Photoacoustic microscopy visualized superficial dermal microvasculature and demonstrated pulsatile signal dynamics. Primarily a feasibility demonstration; diagnostic or clinical usefulness was not established.
20	High-frequency ultrasound (22MHz) in the evaluation of malignant cutaneous neoplasms	Barcaui et al. [2]	2014	Brazil	Text and opinion	BCC, SCC, and melanoma	Not reported	HFUS	Malignant skin neoplasms (BCC, SCC, and melanoma)	HFUS with Doppler is described as providing structural and vascular information that may assist preoperative planning and lesion characterization. Evidence is narrative without quantified microvascular performance or diagnostic accuracy.
21	Region-based SVD processing of high-frequency ultrafast ultrasound to visualize cutaneous vascular networks	Bhatti et al. [6]	2023	Japan	Non-randomized experimental study	Healthy volunteers	Not clearly reported	HFUS	Normal skin	Region-based SVD processing improved visualization of dermal microvessels and subcutaneous vessels compared with standard processing. Promising for enhancing microvascular visibility, but lacks patient data and reference-standard validation.
22	Volumetric visualization of the dermal vasculature with signal and image-based feature extraction on a high-frequency ultrafast ultrasound dataset	Bhatti et al. [37]	2025	Japan	Non-randomized experimental study	Healthy volunteer	1	HFUS	Normal skin	This methodological study used high-frequency ultrafast ultrasound combined with region-based singular value decomposition filtering to visualize dermal and subdermal microvasculature in healthy human skin. The approach enabled 3D visualization of vascular networks and extraction of vessel-related features.
23	High-frequency power Doppler ultrasonography in predicting burn depth: a preliminary case report	Saijo et al. [38]	2024	Japan	Case report	Patients	2	HFUS	Deep fire burns and burn depth assessment	Presence/absence of dermal perfusion on high-frequency power Doppler aligned with tissue viability and burn depth. Potentially useful as an adjunct bedside tool, but evidence is limited to small case data without reference validation.
24	In vivo microcirculation mapping of human skin keloid by 40-MHz ultrafast ultrasound imaging	Chen et al. [39]	2017	Taiwan	Non-randomized experimental study	Volunteer with a keloid	1	HFUS	Keloid (benign dermal fibrosis and microvascular architecture)	Ultrafast Doppler with SVD filtering detected microvascular flow within a keloid scar, demonstrating feasibility of allowing microcirculation mapping. Clinical usefulness is exploratory and needs reproducibility and reference-standard validation.
25	Seeing the unseen with superb microvascular imaging: Ultrasound depiction of normal dermis vessels	Corvino et al. [10]	2022	Italy	Non-randomized experimental study	Healthy volunteers	30	SMI	Normal skin	SMI detected low-flow dermal vessels in all assessed body areas and yielded higher vascular index scores than power Doppler. Useful for demonstrating normal dermal microvasculature and establishing reference vascularity, but evaluated only in healthy subjects.
26	Assessing scleroderma patterns with superb microvascular imaging: is it possible? New prospects for ultrasound	Jasionyte et al. [35]	2023	Lithuania	Case series	Healthy volunteer and patients	4	SMI	Systemic sclerosis (SSc) and Raynaud’s phenomenon	SMI visualized altered nailfold microvascular patterns and avascular areas consistent with systemic sclerosis. Potentially useful as a supplementary real-time tool, but evidence is limited to a very small case series.
27	Human mole contrast-free microvascular imaging using erythrocytes	Salari et al. [12]	2025	Denmark	Case report	Healthy volunteer	1	Super-resolution ultrasound imaging using erythrocytes (SURE)	Moles	They successfully visualized the microvasculature in a human mole and estimated the velocity map and velocity profile of vessels as small as 65 µm in diameter, with flow velocities as low as 3.2 mm/s.
28	Dermatology ultrasound imaging technique, tips and tricks, high-resolution anatomy	Catalano et al. [4]	2020	Italy and Chile	Systematic review	Not reported (review)	Not reported	HFUS, VHFUS, CDI, elastography	The skin in general	Educational review illustrating dermatologic ultrasound techniques, including examples of microvascular imaging (e.g., SMI). Useful for implementation and interpretation guidance, but does not provide new microvascular outcome evidence.
29	Use of new microcirculation software allows the demonstration of dermis vascularization	Catalano et al. [10]	2023	Italy	Non-randomized experimental study	Healthy volunteers	50	SMI and MV-Flow (HFUS, CDI, PDI, and advanced Doppler techniques)	Normal skin (dermal microvascularization)	Advanced Doppler (SMI/MV-Flow) visualized dermal vessels more consistently than power Doppler, which often showed no flow. Clinically useful for improving low-flow dermal microvascular detection, though site- and artifact-sensitive.
30	Shear wave elastography and microvascular ultrasound in response evaluation to calcipotriol + betamethasone foam in plaque psoriasis	Guazzaroni et al. [11]	2021	Italy	Cohort study	Patients	26	MicroV, SWE	Mild to moderate plaque psoriasis, PASI ≥ 4	Microvascular ultrasound showed reduced vascularity in responding plaques and may detect early treatment response alongside SWE. Potentially useful for non-invasive monitoring, but conclusions are limited by small samples and minimal imaging follow-up data.
31	Three-dimensional multistructural quantitative photoacoustic and US imaging of human feet in vivo	Choi et al. [25]	2022	South Korea	Non-randomized experimental study	Healthy volunteers	6	Photoacoustic imaging, US, 3D image reconstruction	Normal skin	3D PA/US provided microvascular images and quantitative parameters (e.g., vessel density/depth) in the foot, including responses to venous occlusion. Useful for peripheral microcirculation assessment, but tested only in healthy subjects without gold-standard comparison.
32	Visualization of skin morphology and microcirculation with high-frequency ultrasound and dual-wavelength photoacoustic microscope	Saijo et al. [28]	2019	Japan	Non-randomized experimental study	Healthy volunteers	40	HFUS and photoacoustic imaging	Normal skin (skin aging and dermal microstructure)	The integrated HFUS/PA system visualized skin structure and microcirculation in 3D and supported functional assessment (e.g., oxygenation-related imaging). Promising for microvascular assessment, but clinical validation metrics are missing.
33	Multimodal ultrasound assessment for monitoring keloid severity and treatment response	Zhou et al. [36]	2025	China	Cohort study	Patients	31	HFUS, SWE, AP, CDFI, PDI	Keloid scars	Significant differences among mild, moderate, and severe keloid groups in thickness, stiffness (SWE parameters), and blood flow (AP mode) (*p* < 0.001). AP imaging was more sensitive than CDFI and PDI in detecting microvascular changes and differentiating keloid severity levels.
34	Morphological aspects of basal cell carcinoma vascularization	Bungărdean et al. [7]	2023	Romania	Review, without systematic methodology	Not reported	3	HFUS and CDI	BCC	Describes typical BCC vascular patterns (often avascular center with peripheral vascularity) and suggests that Doppler may aid characterization. Provides supportive context, but no primary data or validated microvascular outcomes.
35	Advanced multimodal ultrasound for pre-operative assessment of skin tumours: a case series	Kho et al. [40]	2024	Singapore	Case series	Patients	11	B-mode, CDI, SMI, strain elastography, SWE	BCC, SCC	Multimodal ultrasound, especially SMI, revealed tumor-associated microvascular networks and supported preoperative planning. Microvascular depiction appears useful as an adjunct, but evidence is limited to a small observational series.
36	Microvenous reflux in the skin of limbs with superficial venous incompetence	Govind et al. [41]	2018	New Zealand	Case control study	Patients and healthy volunteers	Not clearly reported	SMI and CDI	Chronic venous disease	SMI detected more low-flow microvenous reflux than conventional color Doppler. Improves sensitivity for superficial microvascular abnormalities and may aid earlier assessment, but evidence is limited by sample size and missing variability data.

## Appendix C

**Table A1:** Overview of imaging modalities, systems or platforms, probes or transducers, and reported frequency or software settings across the included studies. Technical equipment reporting was inconsistent across studies, and device-, probe-, and software-level details are therefore only presented when explicitly reported in the source article or extraction material.

Study No	Author and Year of Publication	Modality	System/Platform	Probe/Transducer	Frequency/Software Mode
1	Barcaui et al. 2014 [2]	HFUS	NR	Linear probe	22 MHz
2	Catalano 2020 [4]	HFUS, VHFUS, CDI, Elastography	NR	NR	Review; no single study device
3	Bhatti 2023 [6]	HFUS/ultrafast US	Vantage 256	L38–22v CMUT	31.5 MHz Center frequency
4	Bungărdean 2023 [7]	Review	NR	NR	NR
5	Jaeger 2017 [8]	Combined US + PA	Sigma 5000 IMAGIC	128-element array transducer	Center frequency 7.5 MHz; 40 MHz frequency
6	Catalano 2023 [9]	SMI, MV-Flow	Aplio i800 (Canon) and RS85 Prestige (Samsung Medison)	22-MHz linear probe	NR
7	Corvino 2022 [10]	SMI	Aplio i800 (Canon)	22-MHz linear probe	NR
8	Guazzaroni 2021 [11]	MicroV + SWE	Esaote MyLab 9 (Genova, Italy)	High-frequency linear probe 4–15 MHz	NR
9	Salari 2025 [12]	Super Resolution Using the Erythrocytes	GE Logiq E9 Verasonics Vantage 256^TM^	10 MHz GE L8–18iD	8Mhz; Matlab 2021B
10	Wang 2020 [15]	PAUS	Custom PAUS dermoscope	Bimorph transducer Custom	~33–35 MHz center frequencies
11	Lin 2017 [16]	OR-PAM	Custom OR-PAM	50-Mhz V214-BB-RM (Olympus NDT)	2Hz
12	Favazza 2011 [17]	PAM	NR	V214-BB-RM (Panametrics)	50 MHz Center frequency
13	Ly 2022 [18]	PAM	Dual-fast scanning PAM system (Ohlabs)	Olympus flat transducer	Center Frequency 50 MHz; 200MHz Sampling rate
14	Zafar 2015 [19]	PAI + US	Vevo LAZR (Fujifilm VisualSonics)	256-element linear-array probes	Center Frequencies 15, 21, 40 MHz
15	Zafar 2014 [20]	PAI + US	Vevo 2100 LAZR PAT (VisualSonics)	Linear-array transducer	40 MHz
16	He 2022 [21]	FRSOM/RSOM	Fast RSOM (FRSOM) custom platform	Custom transducer	Reconstructed bands 10–40 MHz and 40–120 MHz;
17	Schwarz 2015 [22]	OAM/RSOM	NR	Ultra-wideband spherical	<25Mhz and between 25 MHz–63.5 MHz and >63.5 MHz
18	Bitton 2007 [23]	Array-based PAM	Custom PAM system	30 MHz linear array (custom)	30 MHz with multichannel receiver system
19	Huynh 2024 [24]	PAT	Custom build PAT scanner (University College London)	Fabry–Perot polymer film ultrasound sensor	0.05–35 MHz
20	Choi 2022 [25]	3D PA/US	E-CUBE 12R (Alpinion medical systems) + Phocus Mobile (Opotek)	128-element L3–12 linear array (Alpinion)	3D foot scanner; 3–12 MHz
21	Zheng 2021 [26]	PAT	Verasonics 128-channel DAQ	L22–8 linear transducer array (Verasonics)	15.6 MHz center frequency
22	Zhang 2019 [27]	PAI	Custom miniaturized PA probe	Planar ultrasound transducer	15 MHz center frequency
23	Saijo 2019 [28]	HFUS + PAM	Custom HFUS + dual-wavelength PAM	Concave Ultrasound transducer	50 MHz center frequency
24	Hsu 2016 [29]	OR-PAM	Custom OR-PAM system	V214, Olympus NDT	50 MHz
25	Heres 2017 [30]	PA imaging + comparator US/PDUS	FULLPHASE prototypecontrolled by MyLab One	SL3323 linear array	7.5 MHz
26	Xu 2016 [31]	PA dermoscopy	Custom PA dermoscope	Integrated custom PA probe	18 MHz Center Frequency
27	Liu 2016 [32]	PAT + OCT + OCTA	Combined multimodal PAT/OCT/OCTA system	Fabry–Perot polymer film ultrasound sensor	NR
28	Rodrigues 2022 [33]	MSOT/optoacoustic imaging	MSOT optoacoustic system, iThera Medical GmbH, Munich, Germany	MSOT measurement probe/3D cup probe	NR
29	Song 2010 [34]	Ultrasound-array photoacoustic microscopy	Custom-built UA-PAM	High Frequency 30 MHz Linear array	30 MHz
30	Jasionyte 2023 [35]	SMI	Canon TUS-AI800, Canon Medical Systems	24 MHz linear transducer	19 MHz
31	Zhou 2025 [36]	CDFI, PDI, AP, SWE	Aixplorer (SuperSonic Imaging)	Linear probe	4–15 MHz
32	Bhatti 2025 [37]	Volumetric HFUS/ultrafast workflow	Vantage 256 High Frequency Configuration, Verasonics	L38–22v, KOLO Medical	30 MHz Center frequency
33	Saijo 2024 [38]	HF Power Doppler	LOGIQ e ultrasound device, GE Healthcare	L10–22-RS	22MHz
34	Chen 2017 [39]	Ultrafast HFUS	Vantage 256 (Verasonics)	MS550D array transducer (Fujifilm VisualSonics)	40 MHz
35	Kho 2024 [40]	B-mode, CDI, SMI, spectral Doppler, SE, SWE	Canon i800 (Canon Medical)	Linear-matrix transducer	24 and 18 MHz
36	Govind 2018 [41]	SMI + color Doppler	Toshiba Aplio 500	Linear array PLB-1005BT transducer	18 MHz

Note: Technical equipment reporting was inconsistent across the included studies. Device-, probe-, and software-level details are summarized only where explicitly reported in the source article or extraction material. Abbreviations: HFUS = high-frequency ultrasound, US = ultrasound, PA = photoacoustic, PAUS = photoacoustic and ultrasound, SURE = super-resolution ultrasound using erythrocytes, SWE = shear wave elastography, SMI = superb microvascular imaging, MV-Flow = microvascular flow imaging, MicroV = microvascular imaging, PD = power Doppler, PDUS = power Doppler ultrasound, CDFI = color Doppler flow imaging, PDI = power Doppler imaging, AP = angioPLUS, PAM = photoacoustic microscopy, OR-PAM = optical-resolution photoacoustic microscopy, PAI = photoacoustic imaging, PAT = photoacoustic tomography, OAM = optoacoustic mesoscopy, RSOM = raster-scan optoacoustic mesoscopy, FRSOM = fast raster-scan optoacoustic mesoscopy, MSOT = multispectral optoacoustic tomography, OCT = optical coherence tomography, OCTA = optical coherence tomography angiography, SVD = singular value decomposition, MEMS = microelectromechanical systems, PVDF = polyvinylidene fluoride, PZT = lead zirconate titanate, DAQ = data acquisition, OPO = optical parametric oscillator, Nd:YAG = neodymium-doped yttrium aluminum garnet, Nd:YLF = neodymium-doped yttrium lithium fluoride, MHz = megahertz, nm = nanometer, NR = not reported, and B-mode = brightness mode.
